# Experimental demonstration of conformal phased array antenna via transformation optics

**DOI:** 10.1038/s41598-018-22165-4

**Published:** 2018-02-28

**Authors:** Juan Lei, Juxing Yang, Xi Chen, Zhiya Zhang, Guang Fu, Yang Hao

**Affiliations:** 1 National Key Laboratory of Antennas and Microwave Technology, Xidian University, Xi’an, 710071 China; 2 School of Electronic Engineering and Computer Science, Queen Mary University of London, London, E1 4NS United Kingdom

## Abstract

Transformation Optics has been proven a versatile technique for designing novel electromagnetic devices and it has much wider applicability in many subject areas related to general wave equations. Among them, quasi-conformal transformation optics (QCTO) can be applied to minimize anisotropy of transformed media and has opened up the possibility to the design of broadband antennas with arbitrary geometries. In this work, a wide-angle scanning conformal phased array based on all-dielectric QCTO lens is designed and experimentally demonstrated. Excited by the same current distribution as such in a conventional planar array, the conformal system in presence of QCTO lens can preserve the same radiation characteristics of a planar array with wide-angle beam-scanning and low side lobe level (SLL). Laplace’s equation subject to Dirichlet-Neumann boundary conditions is adopted to construct the mapping between the virtual and physical spaces. The isotropic lens with graded refractive index is realized by all-dielectric holey structure after an effective parameter approximation. The measurements of the fabricated system agree well with the simulated results, which demonstrate its excellent wide-angle beam scanning performance. Such demonstration paves the way to a robust but efficient array synthesis, as well as multi-beam and beam forming realization of conformal arrays via transformation optics.

## Introduction

Phased array antennas have received increasing attention in recent years for applications in modern wireless communications and radar systems *etc*. due to their flexible beam scan ability^1–4^. A common approach to realize beam scanning is to dynamically control amplitude and phase distributions of array elements. For regular arrays such as ideal linear or planar arrays, such distributions can be achieved by using classical methods of array synthesis, which have been proven to be robust and efficient. However, for conformal antenna arrays mounted on irregular platforms, these design approaches are no longer valid primarily due to the modified antenna radiation of each element. Various optimization methods have therefore been developed to obtain desired amplitude and phase distributions of conformal elements, which are often arbitrary and random.

Both metamaterials and transformation optics (TO) are new emerging technologies which can be used to manipulate electromagnetic waves in unprecedented ways^5^, and have been applied to many engineering designs including conformal array antennas. Initial designs are often based on the use of anisotropic and inhomogeneous media with spatially varying permittivity and permeability, with examples including invisible cloaks, electromagnetic concentrators, field rotators and wave conversion devices^6,7^. For antenna designs, the coordinate transformation medium could be used to manipulate antenna geometries as well as feeding structures^8–10^, which may lead to much improved performance, for instance, to widen beam scanning range of the planar phased array^11,12^.

However practically implementing inhomogeneous and anisotropic media into TO based devices turns out to be complex and challenging, in particular, materials/structures with magnetic responses are often required to achieve good impedance matching and polarization independence. Those materials are often lossy and narrowband due to the inevitably dispersive characteristics of metamaterials. The concept of quasi-conformal transformation optics (QCTO) originally proposed to design the so-called all-dielectric carpet cloak^13^ has been widely adopted in engineering designs. The QCTO mapping can be achieved by solving the inverse Laplace’s equation with sliding boundaries^14^. Although limited to two dimensional configurations with TE polarization, it gives the possibility to implement nonmagnetic devices which can be realized by all-dielectric constituent materials. The QCTO method has hence opened up new horizons in designing electromagnetic devices, especially antennas, and it has been employed to flatten lenses^15–17^, control antenna patterns^18,19^, design conformal antennas^20–24^, and recently, a systematic approach to design a bespoke lens profile based on QCTO for a given excitation is presented in^25^. Among these applications, the theoretical QCTO lens design for transforming between linear and cylindrically conformal arrays is simulated in^21^ and the scanning performance of the conformal array are simulated as well. Although the transformation permittivity distribution is not strictly isotropic and analytic, the lens is nonmagnetic so that it is expected to be broadband. Compared to this design, we focus on the approximation and implementation of the all-dielectric transformed lens with graded refractive index (GRIN), the theoretical analysis and experimental demonstration of the whole antenna-lens system.

In this work, a conformal phased array based on all-dielectric QCTO lens, which can scan radiation beam with low side lobe level (SLL) within wide-angle range, is numerically designed and experimentally demonstrated. Different from the scanning mechanism of Luneburg lens and the beam-scanning control only achieved by the use of transformation media, the method of QCTO lens along with low SLL array synthesis is applied to the design of conformal phased array in order to avoid the complex design and optimization in classical method. The broadband isotropic QCTO lens with GRIN is realized by controlling the density of air holes in dielectric material so that effective material parameters can be accurately matched, the aperture-coupled microstrip antenna array and printed feeding networks are implemented to demonstrate the whole system. The measured far-field patterns of the conformal array with QCTO lens agree well with the simulated ones, which demonstrate its excellent wide-angle beam scanning performance. Through the design of transformation optics, we will avoid the cumbersome procedure which computer optimizations are needed to design conformal phased arrays. We also demonstrate low SLL beam scanning can be achieved within a wide scanning range, even if array elements are fed by a simple scheme with symmetric magnitude and linear phase distributions.

## Results

### Design of the conformal phased array with QCTO lens

Take the conformal cylindrical phased array as an example, the symmetric amplitude and linear phase distributions from planar array synthesis are used as the excitation of conformal phased array surrounded by QCTO lens to achieve low SLL scanning patterns within wide-angle range. The center operating frequency is set to 3.0 GHz and the relative frequency bandwidth is nearly 14%, which is dependent on that of the array element.

**Figure 1:**
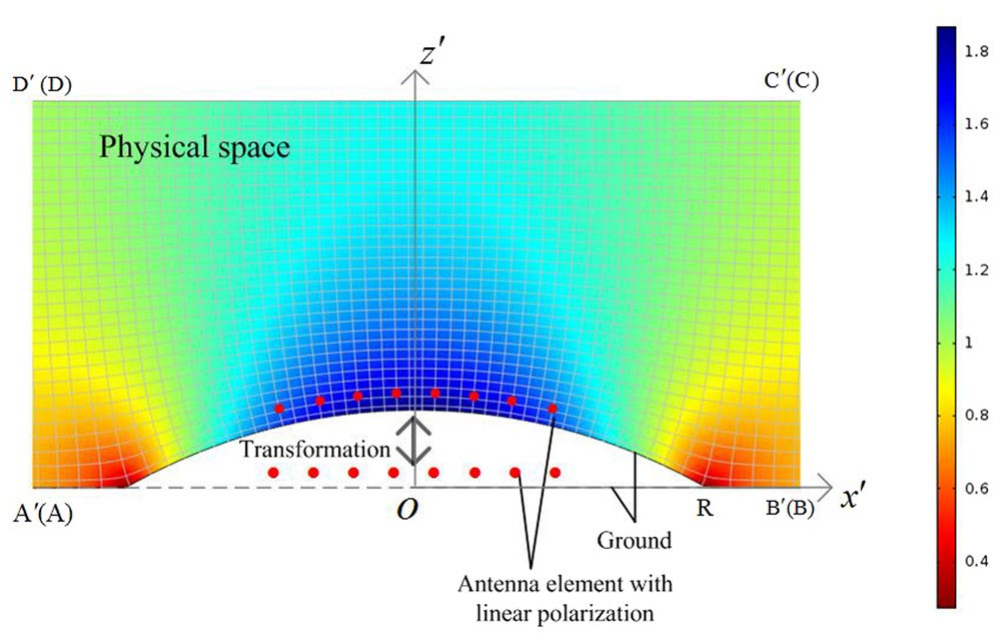
The QCTO mapping from the virtual space (planar dipole array) to the physical space (conformal dipole array with lens) and relative permittivity distribution.

The physical space representing the conformal array with lens in the region A′B′C′D′ is presented in Fig. 1 and transformed from the virtual planar array in the same region ABCD. The two spaces have the same boundaries except segments A′B′ and AB, which are perfect electric conductor boundaries corresponding to the ground shapes of the conformal and planar arrays. The coordinates in the physical and virtual spaces are $(x^{\prime} ,y^{\prime} ,z^{\prime} )$ and $(x,y,z)$ respectively. In the virtual space, the planar array comprises *N* = 8 $\hat{y}$-polarized elements with the array space *d* = 50 mm. The *n*th element located at ${{\bf{r}}}_{{\bf{n}}}=[-(N-1)/2+n]d\hat{x}$ is excited by the current density ${{\bf{J}}}_{{\bf{n}}}={A}_{n}{e}^{j{\phi }_{n}}\hat{y},$ where ${A}_{n}$ and ${\phi }_{n}$ are the magnitude and phase of the *n*th complex current density respectively. The distance between elements and ground is 25 mm. In the physical space, the 8-element conformal array is located on the cylindrically conformal ground, and the original dimensions of the transformed lens are *A′B*′ = *C′D′* = 1000 mm, *A′D*′ = *B*′*C*′ = 400 mm and *OR* = 300 mm, which are miniaturized in the following simulations and measurements according to the relative permittivity approximation of lens. The transformed materials properties of lens are characterized by the dielectric tensors ${\boldsymbol{\varepsilon }}{\boldsymbol{^{\prime} }}$ and ${\boldsymbol{\mu }}{\boldsymbol{^{\prime} }}$. The QCTO mapping is achieved by the solution of Laplace equation subject to Dirichlet-Neumann boundary conditions using COMSOL Multiphysics Partial Differential Equation (PDE) solver, and the minimization of anisotropy allows the lens to be realized using only isotropic electric medium. Dipole antennas convenient for two-dimensional (2D) COMSOL simulations are firstly used as the array elements in the physical and virtual arrays. On the condition of ${\boldsymbol{\mu }}{\boldsymbol{^{\prime} }}={\bf{I}}$, the relative permittivity tensor in the physical space is

1 $${\boldsymbol{\varepsilon }}{\boldsymbol{^{\prime} }}=J{J}^{T}/\det (J)=\varepsilon ^{\prime} {\bf{I}}$$

where $J$ is the Jacobian transformation matrix, and $J=\partial (x^{\prime} ,z^{\prime} )/\partial (x,z)$, **I** is the identity tensor. The computed Cartesian grids in the transformation domain are 600 × 300, which are made sparse for the clear display in Fig. 1. The simulated permittivity distribution of the QCTO lens ranges from 0.3 to 1.9 and values less than unity concentrate around $(\pm R,0)$.

**Figure 2:**
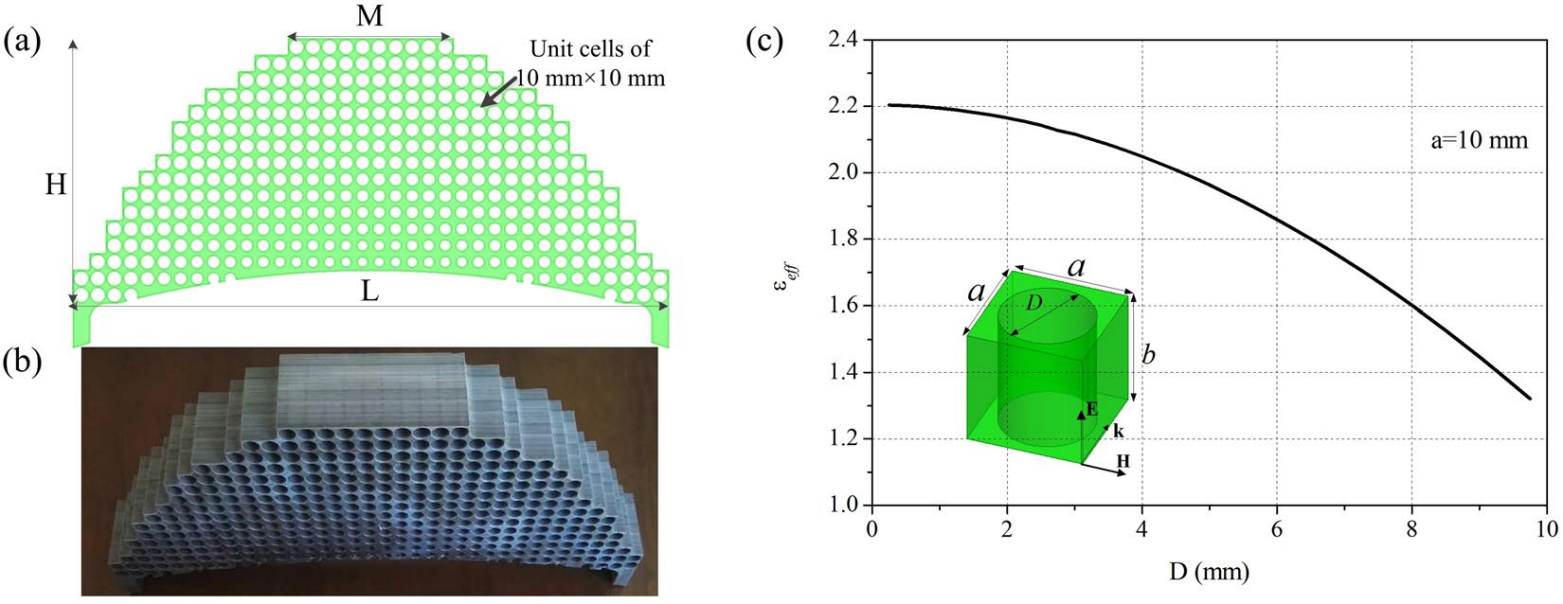
(**a**) Design of the discrete lens, (**b**) Picture of the fabricated lens, (**c**) A parametric analysis is performed to extract the effective permittivity according to the diameter *D* of the drilled-hole with respect to a unit cell with period *a* = 10 mm.

The nonmagnetic lens transformed by QCTO mapping between the virtual and physical spaces is practically designed by the all-dielectric materials after the approximation to relative permittivity lower than 1.3. The discrete lens model shown in Fig. 2(a) has a trapezoidal shape with parameters of *L* = 380 mm, *M* = 100 mm and *H* = 260 mm after discretization. It is realized from a composite drilled-hole unit cells structure which has the advantage of broadband characteristics. According to the effective medium theory, the composite material is considered to be isotropic and homogeneous when the operating wavelength is large enough with respect to the size of unit cell^26^. The respective permittivity of each cell is considered to be constant across the cell and is equal to the average permittivity within the cell. The relative permittivity distribution of the transformed lens ranging from 1.3 to 1.9 is realized from 370 unit cells with the dimensions 10 × 10 mm^2^. The host dielectric medium of the transformed lens, which is presented in Fig. 2(b), has a relative permittivity ${\varepsilon }_{h}=2.2$ and the effective permittivity of each holey dielectric cell can be modified by adjusting the diameter of air holes. A parametric analysis is performed to extract the effective permittivity according to the diameter *D* of air holes with respect to the period *a* of unit cells as shown in Fig. 2(c). The thickness of unit cell *b* is 50 mm in the simulation, which is dependent on the dimension of radiation element, actually, the simulated permittivity of unit cell keeps unchanged as the thickness changes.

**Figure 3:**
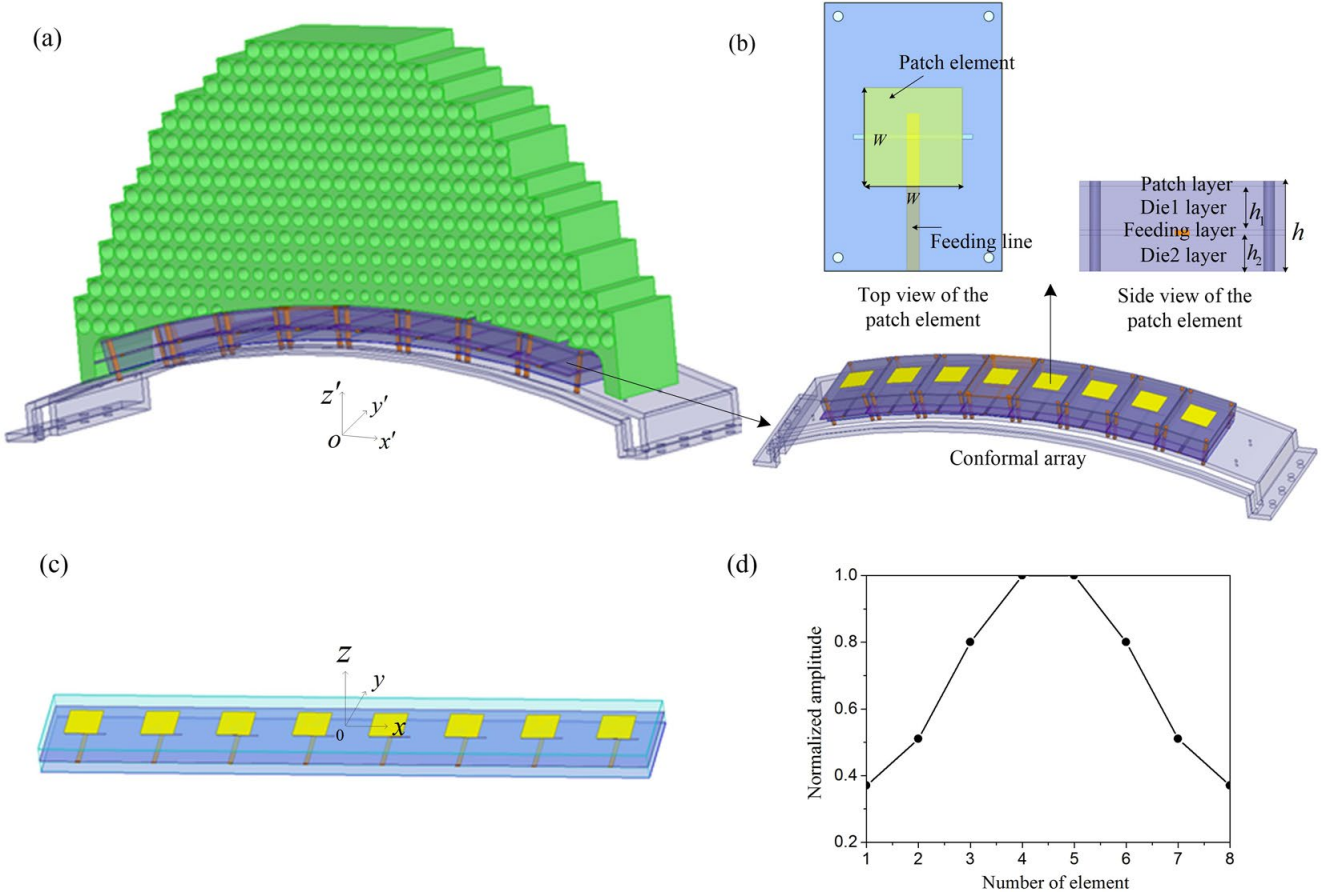
(**a**) The physical conformal patch array with QCTO lens, (**b**) The 8-element conformal patch array and aperture-coupled patch element structure, (**c**) The virtual planar patch array, (**d**) The normalized magnitude distribution synthesized from −25 dB Taylor synthesis.

The designed whole system of conformal phased array covered with QCTO lens is presented in Fig. 3(a), and the conformal array antenna as well as array element structure are shown in Fig. 3(b) in detail. In the practical design, the aperture-coupled microstrip patch antenna array with linear polarization is used as the radiation source because of its wideband characteristic and easy integration with the feeding network. As given in Fig. 3(b), the antenna element is composed of multi-layer dielectric structure with main parameters *W* = 25 mm, *h*_1_ = 8 mm, *h*_2_ = 7 mm and *h* = 17 mm. The performances of phased array antenna including half power beam width (HPBW), main beam direction and SLL are mainly determined by the complex current distribution of antenna elements. In the physical space, the complex current density of the *n*th conformal array element ${{\bf{J}}}_{n}^{/}$, which includes magnitude ${A}_{n}^{/}$ and phase ${\phi }_{n}^{/}$, keeps the same with that of the virtual planar array ${{\bf{J}}}_{n}$ during the transformation, and at the same time the source location of the *n*th element ${{\bf{r}}}_{n}^{/}$ is compressed according to the QCTO mapping, then the conformal phased array in presence of QCTO lens can radiate and scan low SLL beam as if it were a planar one when being excited by the same current distribution with that of virtual planar array.

The electric field generated by the *N*-element virtual planar array presented in Fig. 3(c) can be written as

2 $${\bf{E}}({\bf{r}})=\sum _{n=1}^{N}{{\bf{f}}}_{n}{J}_{n}{e}^{j{k}_{0}{{\bf{r}}}_{n}\cdot {\bf{r}}}=\sum _{n=1}^{N}{{\bf{f}}}_{n}{A}_{n}{e}^{[j({k}_{0}{{\bf{r}}}_{n}\cdot {\bf{r}}+{\phi }_{n})]}$$

where ${{\bf{r}}}_{n}$ and ${{\bf{f}}}_{n}$ represent location and pattern of the *n*th patch element respectively, and ${\bf{r}}$ is the unit propagation vector. The pattern characteristics of the planar array can be modified by adjusting the magnitude and phase distributions of the radiation elements. The magnitude distribution of the virtual planar array is synthesized from the classical Taylor synthesis method for low SLL, which can achieve not only tapered symmetric magnitude distribution but also higher aperture efficiency than other methods such as the Dolph-Chebyshev method *etc*. The normalized magnitude distribution synthesized by −25 dB Taylor synthesis is presented in Fig. 3(d) and the resulted magnitude of the *n*th element satisfies ${A}_{n}={A}_{N+1-n}$. When the main beam directs $({\theta }_{0},{\varphi }_{0})$, the phase ${\phi }_{n}$ of the *n*th element is

3 $${\phi }_{n}=-{k}_{0}{{\bf{r}}}_{{\bf{n}}}\cdot {\bf{r}}=-{k}_{0}({x}_{n}\,\sin \,{\theta }_{0}\,\cos \,{\varphi }_{0}+{y}_{n}\,\sin \,{\theta }_{0}\,\sin \,{\varphi }_{0}+{z}_{n}\,\cos \,{\theta }_{0})$$

where ${k}_{0}$ is the wave number corresponding to the center operating frequency. It can be simplified as ${\phi }_{n}=-{k}_{0}{x}_{n}\,\sin \,{\theta }_{0}$ in the 2D scanning plane, and then the phase difference between two adjacent elements ${\rm{\Delta }}\phi =-{k}_{0}d\,\sin \,{\theta }_{0}$ is a constant.

**Figure 4:**
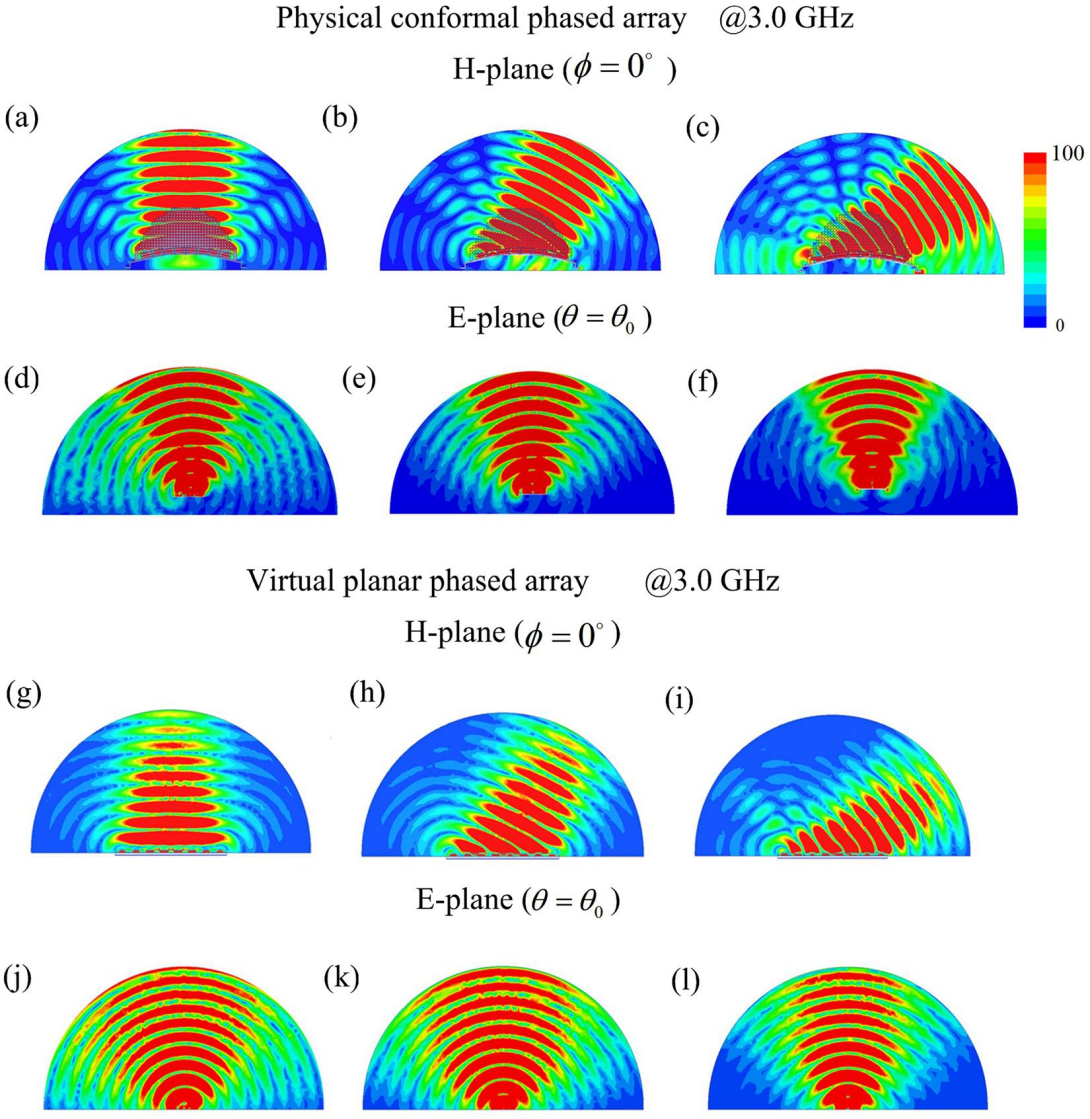
The scanning electric field mappings for the virtual planar and physical conformal arrays with main beam directions at 0°, 25° and 50° respectively at 3.0 GHz (**a**)∼(**c**) physical conformal array (H-plane), (**d**)∼(**f**) physical conformal array (E-plane), (**g**)∼(**i**) virtual planar array (H-plane), (**j**)∼(**l**) virtual planar array (E-plane).

**Figure 5:**
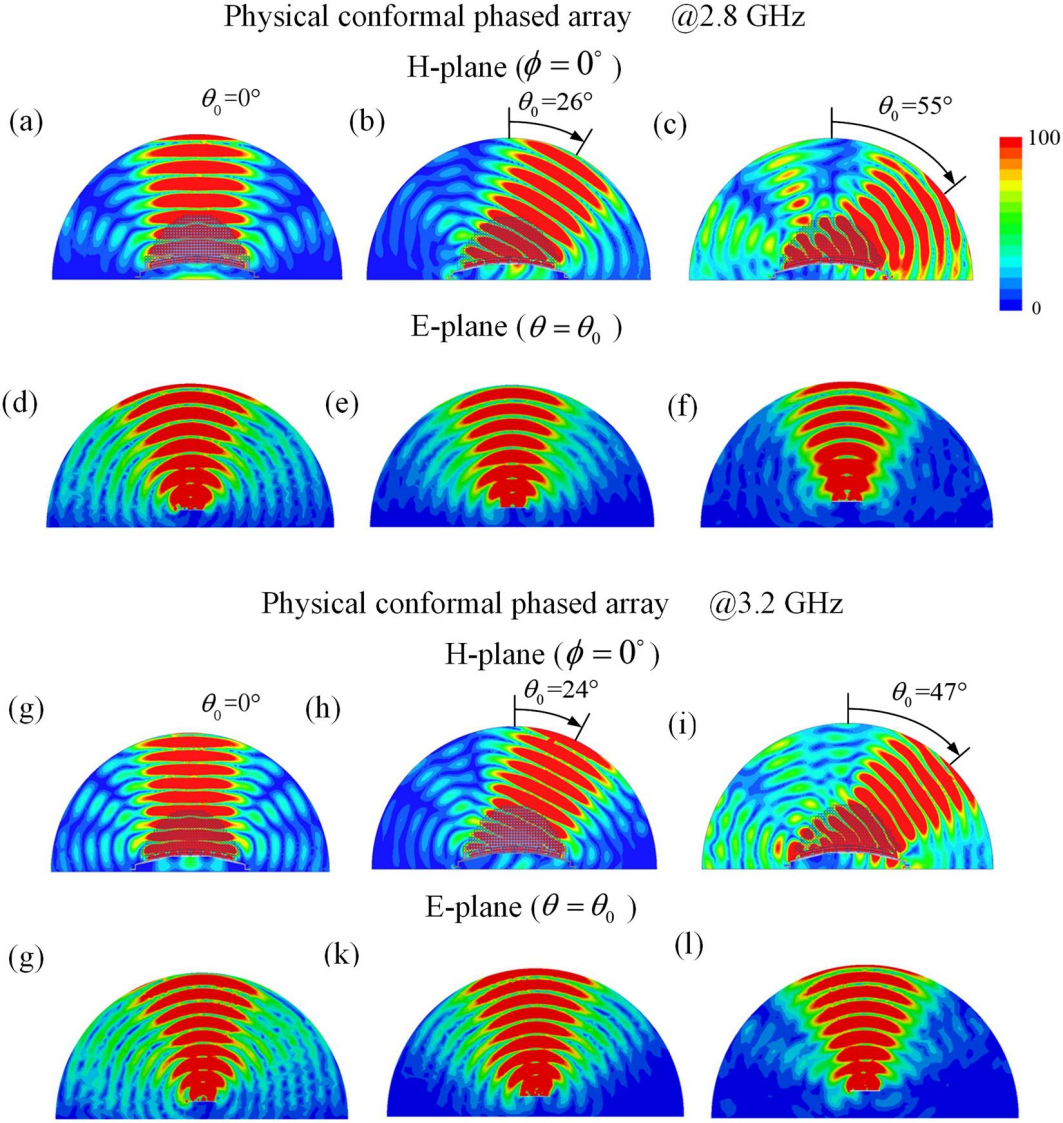
The scanning electric field mappings for the physical conformal array at different frequencies (**a**)∼(**c**) 2.8 GHz (H-plane), (**d**)∼(**f**) 2.8 GHz (E-plane), (**g**)∼(**i**) 3.2 GHz (H-plane), (**j**)∼(**l**)3.2 GHz (E-plane).

The current distribution from Taylor synthesis is used to excite radiation elements of the conformal phased array covered with QCTO lens to achieve low SLL and wide-angle beam scanning. Three different feeding networks, which satisfy the required magnitude and phase distributions with main beam directions at 0°, 25° and 50° respectively, are used to feed the conformal array. Full wave simulations using ANSYS HFSS based on the finite element method are performed to numerically verify the functionality of the all-dielectric holey lens. At the center operating frequency, the simulated H-plane electric field mappings of the physical conformal array with lens are compared with those of virtual planar array at scanning angles of 0°, 25° and 50° in Fig. 4(a~c) and (g~i), and the simulated E-plane mappings of both arrays at different scanning angles are compared in Fig. 4(d~f) and (j~l) respectively. It can be seen that the conformal array with all-dielectric lens can scan radiation beam with low SLL within wide-angle range at H-plane when excited with the tapered symmetric magnitude and linear phase distributions. At a given scanning angle, the beam profile of scanning beam is slightly wider than that of the planar array, which results from approximation to relative permittivity, and the performance obtained from the conformal array with lens also show clearly the enhancements of field intensity at both principal planes, even at the wide scanning angle of 50°, which attribute to the more concentrated electric field distributions at E-plane. Similar H- and E-plane field mappings are also simulated at 2.8 and 3.2 GHz and shown in Fig. 5(a~l) respectively, demonstrating the broadband behavior of the conformal array with lens. It can be clearly observed that the designed lens allows scanning the radiated beam within wide-angle range in the relative band of 14%, corresponding to the operating band of the radiation elements. From the figures, the main beam directions of the scanning beams have little changes of 0°~5° with frequencies since the phase distributions from feeding networks are also frequency dependent.

**Figure 6:**
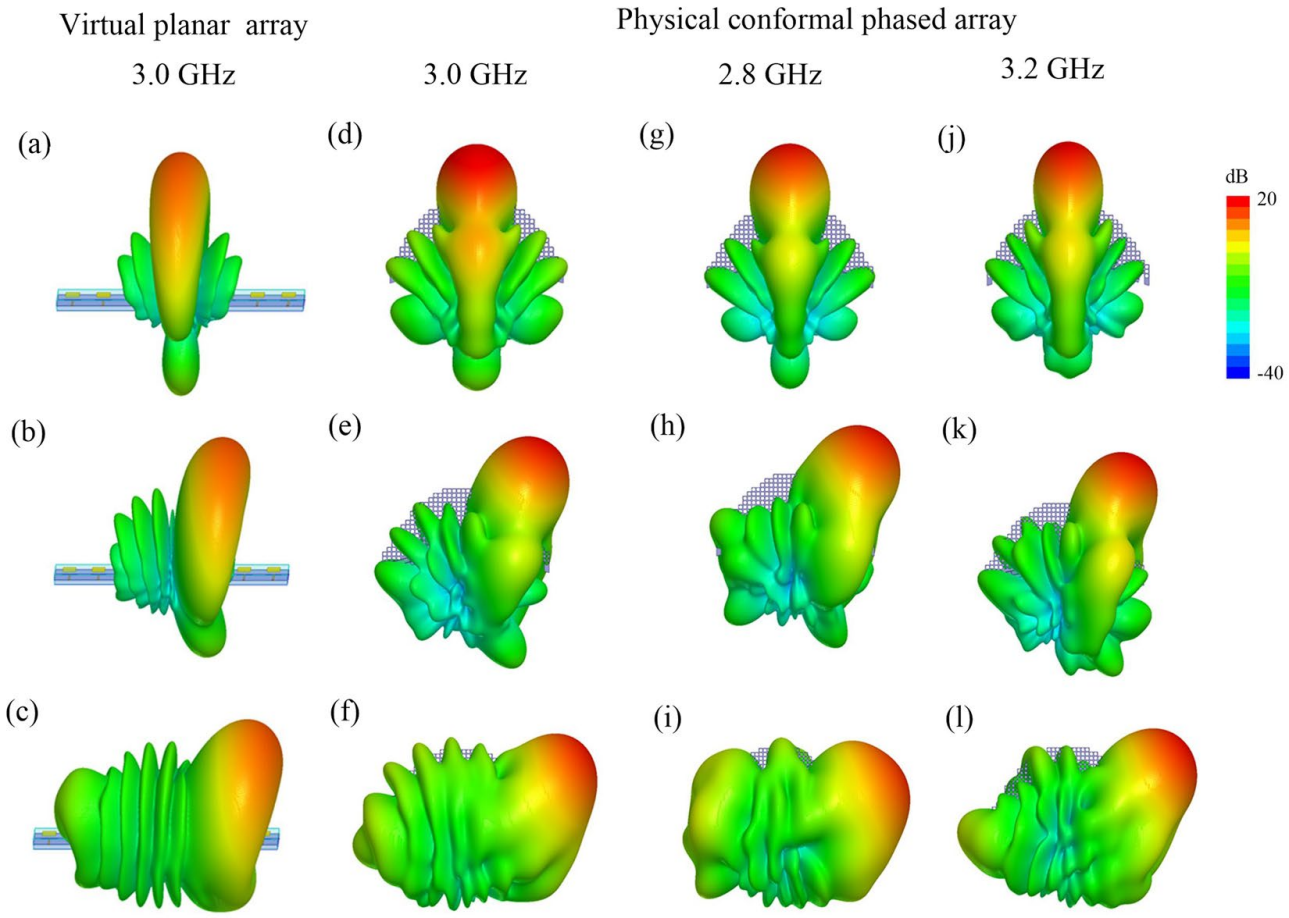
The directivities and 3D patterns for the virtual planar and physical conformal array with main beam directions at 0°, 25° and 50° respectively (**a**)∼(**c**) virtual planar array (3.0 GHz), (**d**)∼(**f**) physical conformal array (3.0 GHz), (**g**)∼(**i**) physical conformal array (2.8 GHz), (**j**)∼(**l**) physical conformal array (3.2 GHz).

The directivities as well as three-dimensional (3D) scanning patterns of virtual planar and physical conformal arrays at the center frequency are presented in Fig. 6(a~f) respectively, the maximum directivities of planar phased array are 13.8~14.4 dB within the wide-angle range of ±50° and the conformal phased array can achieve main lobes with directivities 14.8~16.0 dB, which are 1.0~1.6 dB higher than those of the planar array. As confirmed in the electric field mappings in Fig. 4, the directivity enhancements result from the more concentrated beams at E-plane. The use of lens above the conformal array not only allows restoring linear-phase excitations to scan radiation beams within wide-angle range at H-plane, but also functions as a directivity director at the other principal plane (E-plane). Although the transformed lens realization from all-dielectric materials widens the H-plane beam widths of the scanning beams slightly, it also exhibits dielectric director property which compresses the E-plane beam widths and therefore produces the electric field intensity and directivity enhancements. To demonstrate the directivity and pattern performances of the conformal array with lens within the operating band, similar 3D scanning patterns at 2.8 GHz and 3.2 GHz are presented in Fig. 6(g~l) respectively. The directivity are 13.4~16.7 dB within the operating band and main beam directions which are dependent on the phase distributions of the radiation elements change with the operating frequency.

### Experimental demonstration of the conformal phased array from TO design

**Figure 7:**
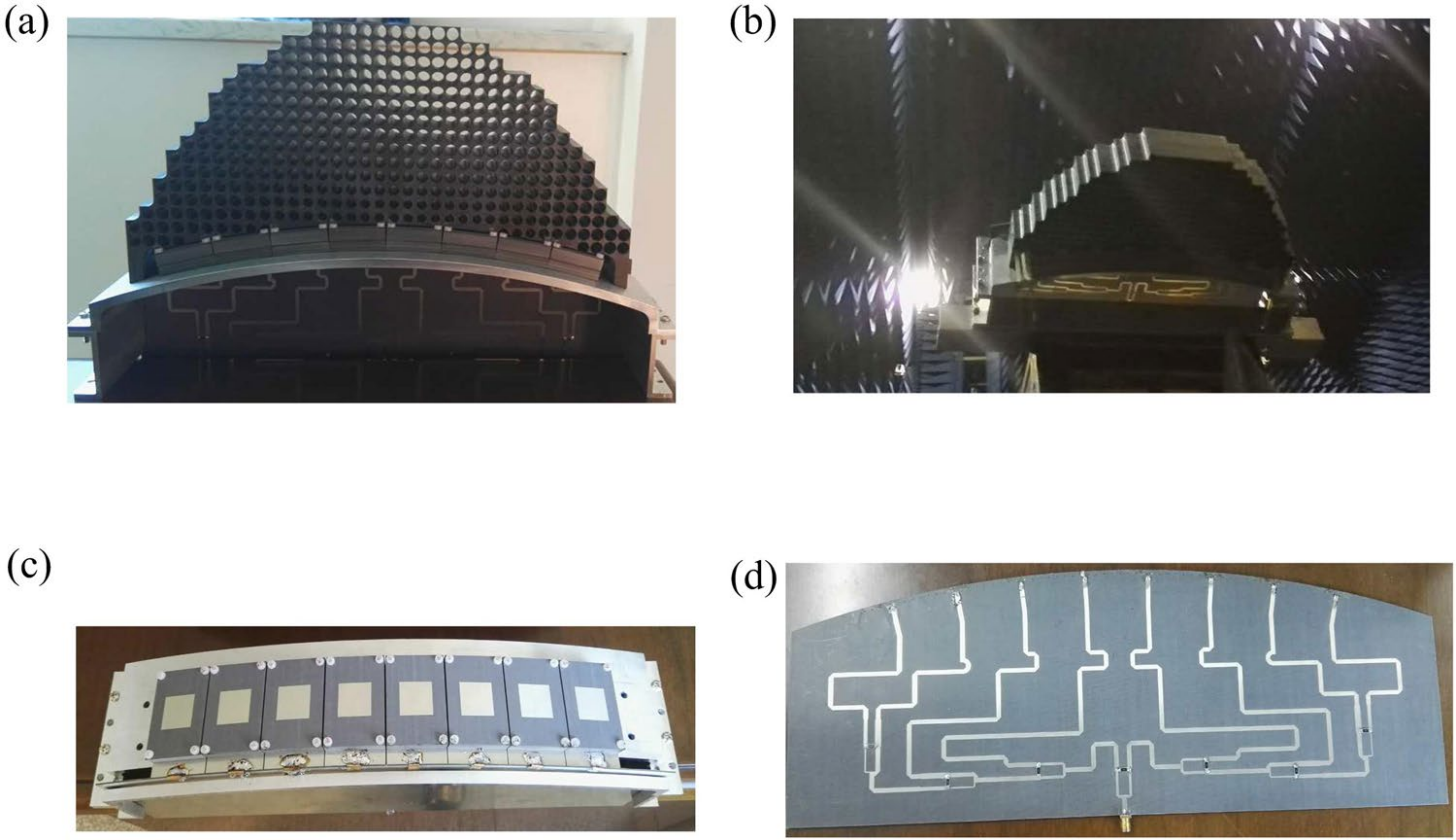
(**a**) The fabricated conformal patch array antenna with lens, (**b**) Antenna in anechoic chamber, (**c**) Conformal patch array antenna, (**d**) The printed feeding network being welded with the radiation elements.

To validate the design approach presented in this paper, the conformal phased array from TO design is fabricated (Fig. 7(a)) and measured (Fig. 7(b)) at microwave frequencies with relative band of 14%. The 8-element aperture-coupled microstrip patch array antenna (Fig. 7(c)) is firstly placed on the cylindrically conformal ground and then covered with the all-dielectric QCTO lens. In the printed feeding network (Fig. 7(d)), the cascaded Wilkinson power dividers are used to achieve accurate and wideband magnitude and phase distributions. The lens is fabricated by using the Computer Numerical Control (CNC) processing with a position accuracy 0.02 mm. The host dielectric has a relative permittivity and thickness of 2.2 and 50 mm respectively. The array elements and feeding networks are printed on Arlon AD255 with a permittivity value of 2.55 and loss tangent of 0.0015. The thickness of the dielectric substrate is 1 mm. The voltage standing wave ratio (VSWR) of the antenna is measured using a Keysight vector network analyzer to demonstrate the band characteristics of the impedance matching. The beam scanning functionality of the lens-based conformal phased array is demonstrated by measuring far-field patterns in the anechoic chamber.

**Figure 8:**
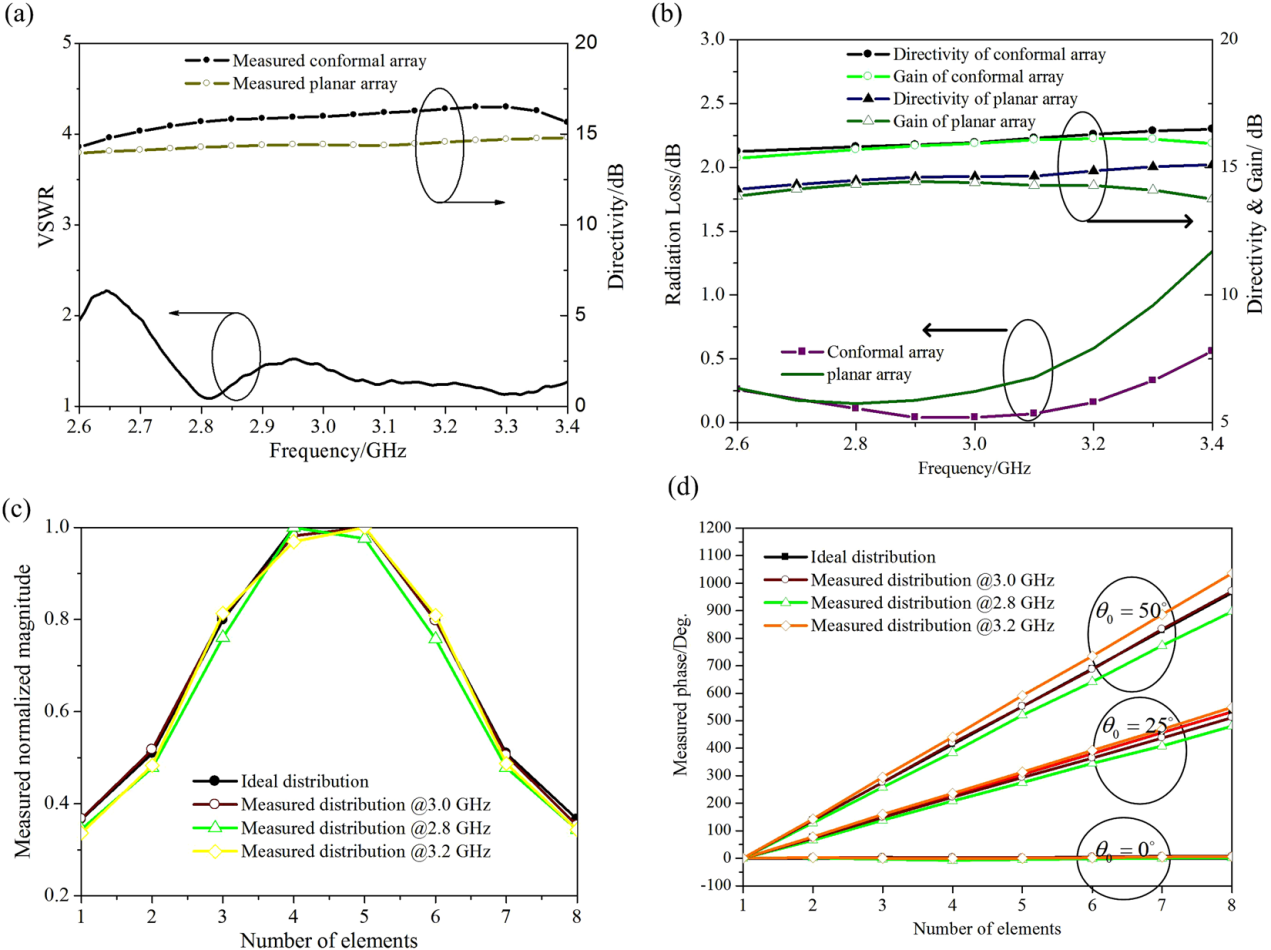
(**a**) The measured VSWR and directivity of the conformal array antenna with lens, (**b**) The radiation loss, antenna directivity and gain, (**c**) The measured normalized magnitude and (**d**) the measured phase.

The measured VSWR of lens-based conformal array is shown in Fig. 8(a), the directivity from integral to the 3D normalized spatial pattern within the frequency band in the broadside condition is also given. At the same time, the directivity of the planar array is measured to quantitatively demonstrate the directivity enhancement of the conformal array with lens. The measured VSWR in the frequency band of 2.8~3.2 GHz is less than 1.5, which indicates that it has excellent impedance matching. The measured directivity of conformal array is 1.6~1.9 dB higher than that of planar array within the tested band at the boresight direction. The radiation losses as well as directivities and gains of both arrays in the broadside condition are presented in Fig. 8(b). From the comparison of antenna directivity with gain, the conformal array with lens has a radiation loss of 0.15~0.25 dB, which is 0~0.4 dB lower than that of the planar array and further indicates the lens’s ability to control the scanning beams with high efficiency in the broad band. The application of lens can broaden the frequency band of conformal array antenna, which decreases the antenna reflection loss, and moreover, the radiation efficiency of array can be increased by the covering of lens because of the improved current distribution of patch elements. The insert loss of the feeding network is not included in the analysis for a clearer explanation and the simulated insert loss of the network is 0.4~0.6 dB within the test band. As for the additional loss of the transformed lens, we think that it is dependent on the loss tangent of lens host dielectric, which is 0.002 in this work. The loss from lens is lower than 0.15~0.25 dB within the test band because of the low loss property of host dielectric. The measured normalized magnitude and phase distributions from feeding networks at typical frequencies 2.8, 3.0 and 3.2 GHz presented in Fig. 8(c) and (d) are entirely consistent with the theoretical results, which therefore ensure the scanning functionality of the conformal array with lens.

**Figure 9:**
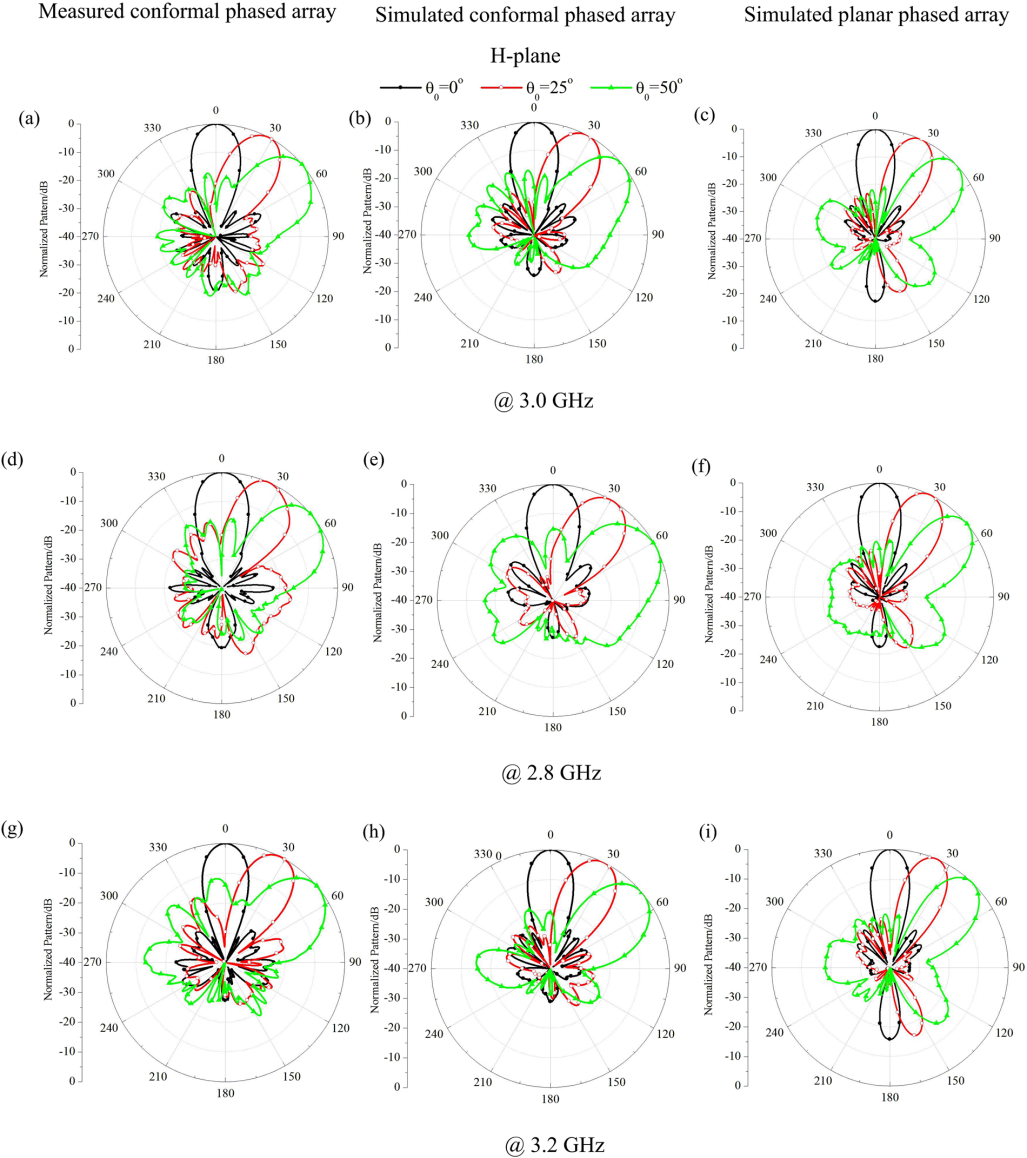
The measured and simulated H-plane far-field scanning patterns for the planar and conformal arrays (**a**)∼(**c**) 3.0 GHz, (**d**)∼(**f**) 2.8 GHz, (**g**)∼(**i**) 3.2 GHz.

**Figure 10:**
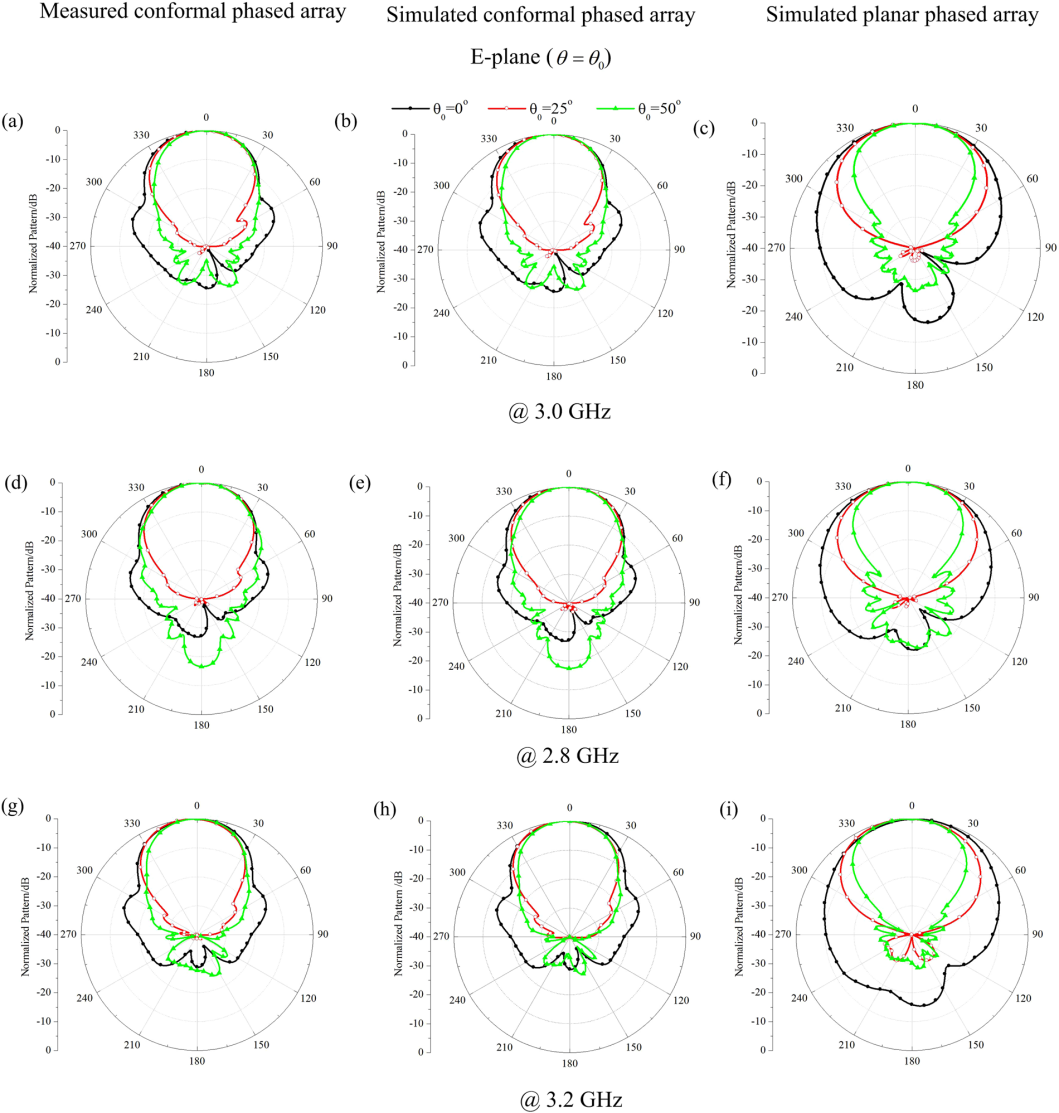
The measured and simulated E-plane far-field scanning patterns for the planar and conformal arrays (**a**)∼(**c**) 3.0 GHz, (**d**)∼(**f**) 2.8 GHz, (**g**)∼(**i**) 3.2 GHz.

The measured far-field H-plane scanning patterns with different main beam directions for the conformal antenna array with lens at 2.8, 3.0 and 3.2 GHz are presented in Fig. 9. Full-wave simulations of the far-field patterns through HFSS have also been carried out to verify the performance of the presented array-lens system, and at the same time, corresponding patterns of planar array are also simulated for comparison. Figure 9(a~c) show the measured and simulated H-plane scanning patterns of both arrays, and as confirmed in the electric field mappings and 3D radiation patterns, the conformal array in presence of lens has wide-angle scanning beam characteristic with low SLL at H-plane, which assembles the performance of the virtual planar array except the little broadening of HPBW. From those figures, measured beam directions and HPBWs agree well with simulated ones, the measured SLLs are consistent with the simulated ones and 0.0~2.5 dB higher than −25 dB within the scanning range of 25°. For the wide-angle of 50°, both the measured and simulated maximum SLLs rise to −17 dB. This is partly due to the mutual coupling between array elements, which becomes even stronger at large scanning angle, and another aspect of the SLL rise attributes to the approximation of permittivity distribution lower than 1.3, which has more adverse effect on scanning beams at large angles. It is also illustrated from the figures that the proposed conformal array with lens can achieve wide angle scanning beam with low SLL in a relative band of nearly 14%. At the low frequency of 2.8 GHz, the HPBWs and main beam directions become wider and higher than those of planar array at the center frequency, and at the high frequency of 3.2 GHz, the HPBWs and main beam directions become narrower and lower. This is because of the changes of element space determined by different wavelengths. The measured and simulated corresponding E-plane far-field patterns of the conformal array with lens are presented in Fig. 10, which are also compared with those of the virtual planar array at different scanning angles. The measured patterns agree well with the simulated ones at E-plane and have narrower HPBWs than those of the planar array, which are consistent with the electric field mappings and 3D radiation patterns, and further demonstrate the directivity director functionality of the QCTO lens. The performance comparison of the planar array with conformal array in presence of lens are given in Table 1 in detail.

**Table 1:** Performance comparison of the planar array with conformal array in presence of lens.

	Planar phased array			Conformal phased array with lens					
	Simulated			Simulated			Measured		
Frequency/GHz	3.0								
Main beam direction/°	0	25	48	0	25	50	0	25	50
Directivity/dB	14.4	14.3	13.8	16.0	15.9	14.8	15.8	15.9	14.7
HPBW_H_/°	15.4	17.0	19.9	18.7	20.2	23.65	19.5	19.3	26.6
SLL_H_/dB	−24.9	−23.2	−15.4	−24.3	−23.2	−17.0	−22.1	−21.5	−17.0
HPBW_E_/°	82.1	67.4	52.0	51.8	46.5	41.4	49.5	45.5	41.5
Frequency/GHz	2.8								
Main beam direction/°	0	26	52	0	26	55	0	26	54
Directivity/dB	14.3	14.0	14.1	15.5	15.2	13.4	15.8	15.2	13.6
HPBW_H_/°	16.4	18.39	20.1	20.0	22.0	32.2	20.5	21.0	30.1
SLL_H_/dB	−25.3	−23.4	−13.5	−20.9	−21.3	−13.0	−22.2	−16.6	−15.5
HPBW_E_/°	82.0	67.9	52.1	50.1	47.8	41.2	50.7	47.5	42.9
Frequency/GHz	3.2								
Main beam direction/°	0	24	46	0	24	47	0	24	47
Directivity/dB	14.6	14.2	14.1	16.7	16.1	15.6	16.3	16.1	15.1
HPBW_H_/°	14.5	15.8	19.3	17.0	20.1	24.1	16.9	20.0	24.4
SLL_H_\/dB	−24.7	−23.5	−14.5	−24.3	−23.5	−15.0	−23.8	−16.1	−11.7
HPBW_E_/°	80.0	67.8	53.7	44.7	45.7	41.6	49.7	45.2	41.5

## Discussion

**Figure 11:**
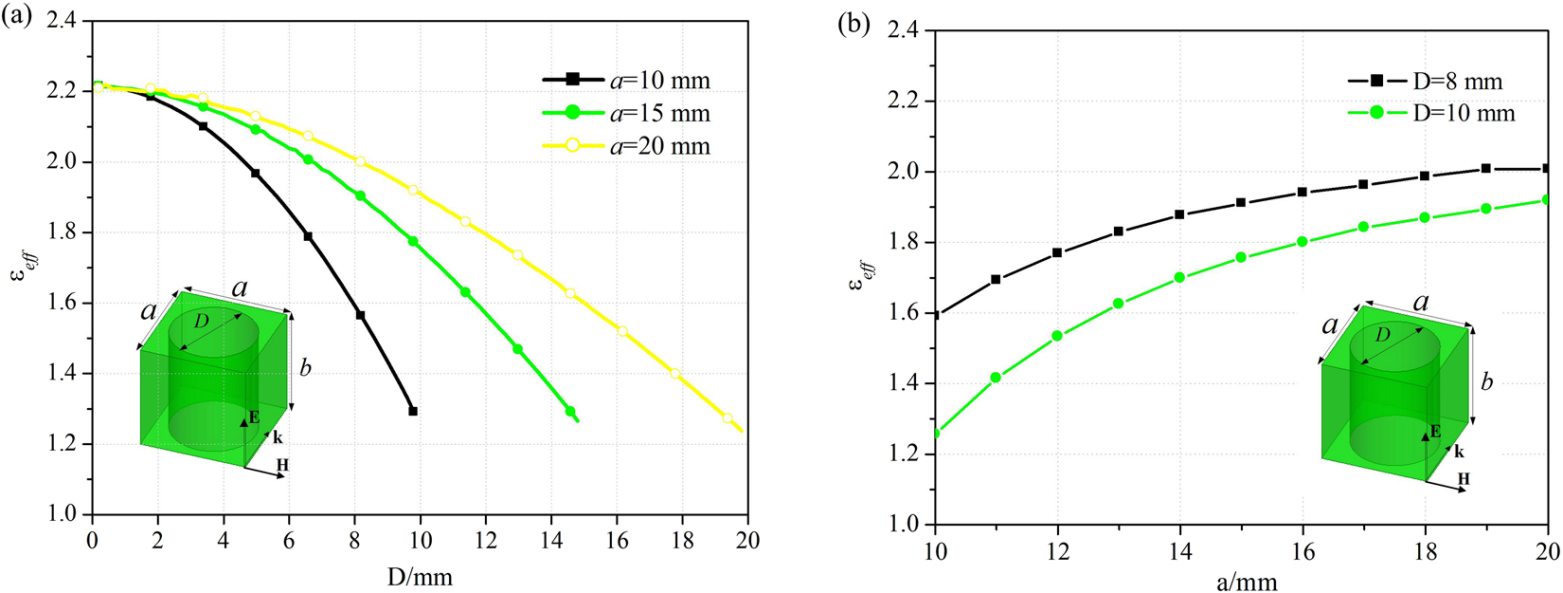
The effective permittivity analysis (**a**) according to the diameter *D* of the drilled-hole with respect to a unit cell with period *a* = 10, 15 and 20 mm, (**b**) according to the unit cell dimension *a* with respect to diameter *D* = 8 and 10 mm respectively.

The conformal array with all-dielectric lens can scan radiation scanning beam with low SLL within wide-angle range when excited with the current distribution from Taylor synthesis. Although the approximation to relative permittivity lower than 1.3 broadens the HPBWs at H-plane slightly, the conformal array with lens shows clearly the enhancements of electric field intensity and antenna directivity at all scanning angles, which attribute to the HPBW compressions at E-plane. It is emphasized that the QCTO method is only valid at 2D space of H-plane and the uniform dielectric distribution of the lens along the polarization direction of radiation source functions as a common lens which has ability to bunch the radiation beams. For the realization of the all-dielectric lens with GRIN, periodic unit cells with different holes are adopted to realize the transformed inhomogeneous permittivity distribution in this work. In fact, different design methods such as changeable unit cells and holes, or fixed holes and changeable unit cells can be used to achieve the GRIN lens^27,28^, which are also simulated and presented in Fig. 11(a) and (b) respectively. However the relative permittivity of the lens is truncated at about 1.25 in all the designs, for example, when the extreme case of *a* = 10 mm, *D* = 10 mm shown in Fig. 11(a) is considered, the realized effective permittivity is 1.25. Taking the practical fabrication accuracy and structure intensity into account, the transformed lens with permittivity ranging from 1.3 to 1.9 is designed and implemented, and its graded refractive index can reduce the energy reflection and achieve good impedance matching between the lens and free space, which can also be observed from the continuous electric field mappings shown in Fig. 4.

**Figure 12:**
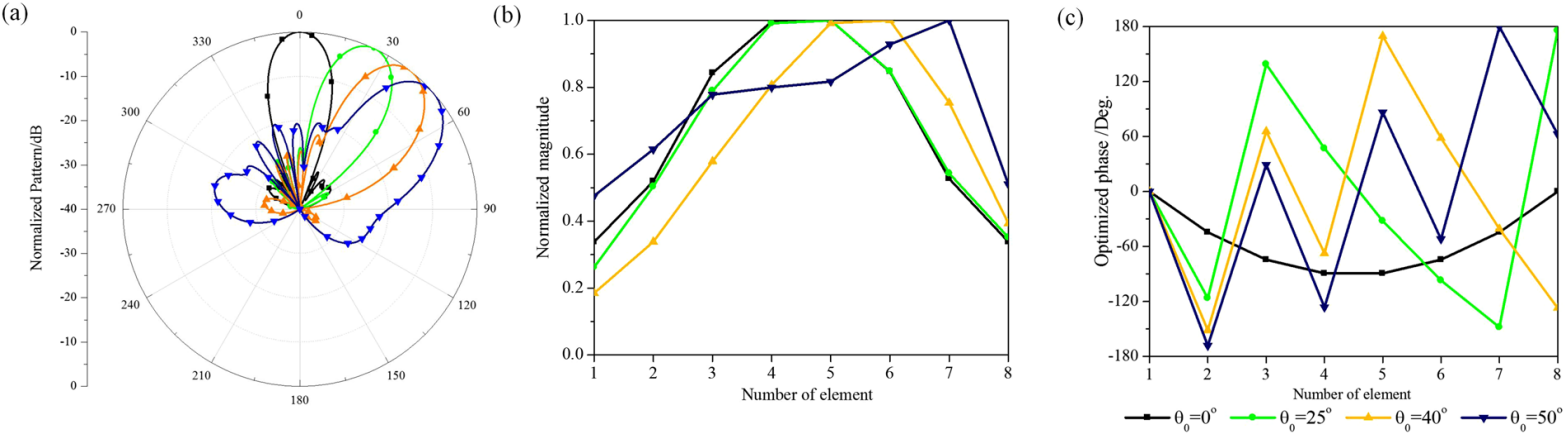
The optimized performances of the conventional conformal array at 3.0 GHz (**a**) far-field scanning patterns (**b**) normalized magnitude distributions and (**c**) phase distributions.

The frequency bandwidth of the presented conformal phased array with lens can be broadened by using wideband radiation elements and feeding networks as the all- dielectric lens is inherently broadband. The relative bandwidth is nearly 14% in our demonstration. And, through the design of transformation optics, the cumbersome procedure which optimization methods are needed to design conformal phased arrays can be avoided and higher directivity can be achieved. In this work, the conventional 8-element conformal phased array shown in Fig. 1 (without lens), which has different element patterns due to the conformal ground, is also simulated for comparison. A global particle swarm optimization (PSO) method is used to optimize the conformal array, and it aims to find the optimal current distribution of radiation elements which minimizes the difference between the antenna and desired patterns. The scanning patterns of the conventional conformal array with the main beam directions 0°, 25°, 40° and 50°, as well as magnitude and phase distributions at the center frequency are presented in Fig. 12(a~c) respectively. It can be seen that the conventional conformal array can realize the wide-angle scanning beams with low SLLs after optimization. At the center frequency, the simulated directivities are 1.2~2.8 dB lower than those of the conformal array with lens within the scanning range of ±50°. The radiation loss within the test band is lower than 0.5 dB. Moreover, compared with the symmetric magnitude and linear phase distributions in the design of conformal array via transformation optics, the optimal magnitude and phase distributions of conventional conformal array are arbitrary and changed with the scanning angles, which are more difficult to be implemented and limit the operating bandwidth of the feeding networks to some extent.

In conclusion, we have designed a wide-angle scanning conformal phased array with low SLL via transformation optics, which is realized through all-dielectric lens. The use of practical lens above the conformal array allows restoring not only the in-phase excitation to create broadside beam, but also the linear phase distribution to achieve the scanning beam within wide scanning range. The measured far-field patterns demonstrate the low SLL and wide angle scanning performances of the conformal phased array with lens within a relative frequency bandwidth of nearly 14%. We also demonstrate that low SLL wide-angle beam scanning can be achieved even if array elements are fed by a simple current distribution from classical Taylor synthesis. Such experimental demonstration presents a remarkable degree of advance for fast and efficient array synthesis, beam forming and multi-beam realization of conformal arrays via transformation optics.

## Methods

### Experiemental setup

The measurement was carried out by two steps. Firstly, the current magnitude and phase distributions of the conformal array from feeding networks are measured using the Keysight vector network analyzer, and then the far-field patterns of the conformal phased array with lens at principal planes are measured in the anechoic chamber.

### Numerical simulation

Numerical simulations including designs of QCTO mapping and relative permittivity approximation are performed using the COMSOL 2D RF module. The final conformal array antenna design with physically realized holey dielectric lens is performed by full wave simulation through AYSYS HFSS.
